# Promoting social plasticity in developmental disorders with non-invasive brain stimulation techniques

**DOI:** 10.3389/fnins.2015.00294

**Published:** 2015-09-01

**Authors:** Paulo S. Boggio, Manish K. Asthana, Thiago L. Costa, Cláudia A. Valasek, Ana A. C. Osório

**Affiliations:** Social and Cognitive Neuroscience Laboratory and Developmental Disorders Program, Center for Health and Biological Sciences, Mackenzie Presbyterian UniversitySao Paulo, Brazil

**Keywords:** brain stimulation, neuromodulation, developmental disorders, autism, Williams syndrome, schizophrenia, social cognition

## Abstract

Being socially connected directly impacts our basic needs and survival. People with deficits in social cognition might exhibit abnormal behaviors and face many challenges in our highly social-dependent world. These challenges and limitations are associated with a substantial economical and subjective impact. As many conditions where social cognition is affected are highly prevalent, more treatments have to be developed. Based on recent research, we review studies where non-invasive neuromodulatory techniques have been used to promote Social Plasticity in developmental disorders. We focused on three populations where non-invasive brain stimulation seems to be a promising approach in inducing social plasticity: Schizophrenia, Autism Spectrum Disorder (ASD) and Williams Syndrome (WS). There are still very few studies directly evaluating the effects of transcranial direct current stimulation (tDCS) and transcranial magnetic stimulation (TMS) in the social cognition of these populations. However, when considering the promising preliminary evidences presented in this review and the limited amount of clinical interventions available for treating social cognition deficits in these populations today, it is clear that the social neuroscientist arsenal may profit from non-invasive brain stimulation techniques for rehabilitation and promotion of social plasticity.

## Introduction

One of the crucial aspects of human beings is the urge to be socially connected. From the need to be cared for (newborn babies) to the pleasure of being in a romantic relationship, being socially connected directly impacts our basic needs and survival. People with deficits in social cognition might present abnormal behaviors in our highly social-dependent world. Along this line, a lot of effort has been allocated to studies on neuropsychiatric disorders. More specifically, several studies have attempted to understand the role of social cognition in developmental disorders by examining their behavioral manifestations. Also, electrophysiological and neuroimaging studies have taught us about the functional and structural neuronal basis of different developmental disorders.

We have also been learning about brain plasticity—from the good, bad, and ugly. The negative impact of lesions and the positive impact of enriched environments on cognitive functions are few examples of it. Based on recent research, we propose a review of the use of non-invasive neuromodulatory techniques to promote Social Plasticity (i.e., the modulation of the neural substrate associated with social cognition aiming for more adaptive social interactions) in some developmental disorders. Particularly, we will focus on Transcranial Magnetic Stimulation (TMS) and Transcranial Direct Current Stimulation (tDCS).

The impact of social cognition alterations or deficits faced by some clinical populations is well known. Nonetheless, most research on non-invasive brain stimulation techniques in the treatment and rehabilitation of clinical populations has focused on other aspects of cognition (such as executive functions, attention, or memory). In this scenario, such techniques might also play a crucial role in addressing impairments in social cognition and behavior, as we will argue below. As social neuroscience is a relatively new field and the use of non-invasive brain stimulation techniques in this context is even more recent, it should come as no surprise that only very few works have used these techniques to approach these issues. In this paper we will emphasize three populations where non-invasive brain stimulation seems to be a promising technique in inducing social plasticity: Schizophrenia, Autism Spectrum Disorder (ASD) and Williams Syndrome (WS). Our main goal is to emphasize how non-invasive brain modulation may be a valuable strategy in this context and how the plasticity of social cognition and behavior in clinical populations is a possible and relevant goal for contemporary research.

## Autism spectrum disorder

There are certainly more investigations of social cognition in ASD than in schizophrenia and WS and it seems fair to say (although a systematic comparison is lacking) that there are more numerous and more effective interventions for social cognition in ASD than in the other two disorders. A review of therapies available for children with ASD can be found in Warren et al. (2011). Among the recent and still experimental ones we highlight intranasal oxytocin and the Early Start Denver Model (all reviewed in Canitano, 2014). Intranasal oxytocin has been shown to increase patients' gaze fixation on the most socially informative regions of the face and to increase interactions with partners and feelings of trust in an experimental paradigm (Andari et al., 2010). In fact, a systematic review of seven studies evaluating oxytocin interventions in ASD found significant effects in all studies but one (Preti et al., 2014). A behavioral technique that has also showed promising results is the Early Start Denver Model, an applied behavior analysis based educational intervention focused on building communicative abilities, enhancing social attention and reinforcing social interaction (e.g., Fulton et al., 2014).

These interventions and results show that it is indeed possible to induce plasticity of social functioning in ASD patients. Nonetheless, ASD expression is very heterogeneous, comprising a whole spectrum of different symptoms in different severities. The outcomes of possible interventions tend to be very variable too (e.g., Warren et al., 2011; Magiati et al., 2014; Preti et al., 2014). Also, side effects of medical interventions might be significant (e.g., Warren et al., 2011). In this context new interventions must be developed, including combinations of complementary strategies. Until now, few studies involving neuromodulation techniques in patients with autism have been conducted and most part used Transcranial Magnetic Stimulation (TMS) (see Table 1).

**Table 1:** **Summary of parameters and results for papers employing TMS or tDCS in developmental disorders**.

**References**	**Condition and sample size**	**Age**	**Stimulation Parameters**	**Results**
Théoret et al., 2005	ASD = 10	23–58	One session of single-pulse TMS and ppTMS over M1^*^.	Cortical excitability in ASD group was significantly lower.
				No group difference in RMT^*^ or response to ppTMS.
Enticott et al., 2012	ASD = 34	M = 26.32	One session of single-pulse TMS over M1^*^.	Observation of single static hand stimuli don't induce difference in degree of coticospinal excitabilitay in both groups.
Minio-Paluello et al., 2009	ASD = 16	M = 28	One session of single-pulse TMS over M1^*^.	Observation of painful movements don't induce corticospinal modulation in ASD group.
Puzzo et al., 2009	ASD = 20	ASD—low traits group: M = 23.7	One session of single-pulse TMS over M1^*^.	No difference between the groups regarding the images in White screen was observed. In observation of actions videos compared to static images participants with low traits of autism exhibited higher MEP amplitudes. This result wasn't observed in high traits of autism group.
		ASD—high traits group: M = 24.5		
Sokhadze et al., 2009	ASD = 8	12–27	rTMS- 150 pulses at 0.5 Hz and 90% RMT^*^ over left DLPFC^*^ twice per week during 3 weeks.	Normalization in event-related potencial—P300 and was induced gamma frequency eletroencephalography activity. In addition, was observed a reduction in ritualistic behavior.
Sokhadze et al., 2010	ASD = 13	9–27	rTMS- 150 pulses at 0.5 Hz and 90% RMT over left DLPFC^*^ twice per week during 3 weeks.	The post-tests scores showed a reduction in repetitive-ritualistic behavior in the stimulated.
Baruth et al., 2010	ASD = 16	9–26	rTMS- 150 pulses at 1 Hz and 90% RMT over left DLPFC once per week during 6 weeks. The same protocol was applied over the right DLPFC^*^.	Improvement in repetitive behaviors and irritability reported by their caregivers.
Casanova et al., 2012	ASD = 25	9–19	rTMS- 150 pulses at 1 Hz and 90% RMT over left DLPFC^*^ once per week during 6 weeks. The same protocol was applied over the right DLPFC.	The results showed better accuracy on the selective attention task, improvements in ERP index of visual processing and improvement in repetitive behaviors and irritability reported.
Sokhadze et al., 2012	ASD = 20	9–21	rTMS- 150 pulses at 1 Hz and 90% RMT over left DLPFC^*^ once per week during 6 weeks. The same protocol was applied over the right DLPFC.	The post-test showed improvement in ERP index and better performance in the error monitoring task.
Enticott et al., 2014	ASD = 28	18–59	Deep bilateral rTMS over DMPFC^*^ at 5 Hz during 15 min for 15 consecutive days.	Significant reduction in social relating symptoms. Significant reduction in self-oriented anxiety during difficult and emotional social situations.
Sokhadze et al., 2014	ASD = 27	9–21	rTMS—18 sessions of 1 Hz over DLPFC^*^, combined with neurofeedback.	Results showed modulation/normalization of many electrophysiological markers and behavioral reaction during executive function test and a decrease in social withdrawal scores.
D'Urso et al., 2015	ASD = 12	18–26	Ten daily sessions of cathodal tDCS over left DLPFC^*^—20 min/1.5 mA.	Forty-five percentage decrease in social withdrawal scores and a 58% decrease in hyperactivity (as measured in the ABC scale).
Brunelin et al., 2012	Schizophrenia = 30	M = 40.4	Two daily sessions of tDCS for 5 days. Anodal placed over the left DLPFC^*^ and cathodal over the temporal cortex.	Decreased severity of auditory hallucinations and negative symptoms after tDCCS.
Mehta et al., 2014	Schizophrenia = 54	Antipsychotic naïve patients: M = 33.6; Medicated patients: M = 29.19	Single pulse and paired-pulse TMS over the motor cortex to assess intracortical inhibition.	Deficits in intracortical inhibition in a sample of antipsychotic naïve patients. This deficits are correlated with social cognition scores.
Palm et al., 2013	Schizophrenia case study	19	Ten daily tDCS sessions. Anodal placed over the left DLPFC^*^ and cathodal over the temporal cortex.	Reduction in hallucination frequency, positive and negative symptoms after tDCS. Changes in functional connectivity observed in resting state fMRI.

* *RMT, Resting motor threshold; M1, Primary motor cortex; DLPFC, dorso lateral prefrontal cortex; DMPFC, dorso medial prefrontal cortex*.

Some studies with TMS focus on motor functions, taking into account assumptions about partial impairment of the mirror neuron system (MNS). The MNS is responsible for understanding intention and imitation of behavior observed in other subjects. In addition to being involved in language development (Williams et al., 2001).

Studies with observation and/or mentoring of motor actions have been used to study the functions of the MNS. From the studies conducted in healthy volunteers (Fadiga et al., 1999; Fourkas et al., 2006; Cesari et al., 2011), it can be seen that TMS provides accurate measurements of cortical excitability and confirms the increased activity as well as motor threshold reduction in motor cortical areas during observation and mental imagery related to motor function. Considering these data, some authors have investigated the relationship between motor imagery and cortical excitability in ASD. Théoret et al. (2005) showed that cortical excitability in autism is significantly lower compared to a control group during a finger movement observation task. The observation of the movements of the fingers facilitated motor evoked potential generated by TMS in the control group but not in the autism group. Other studies with single-pulse TMS show that the observation of specific motor movements or observations of painful movements do not induce corticospinal modulation in patients with ASD (Minio-Paluello et al., 2009; Enticott et al., 2012). Similar results were observed by Puzzo et al. (2009). After measurement of motor evoked potentials MEPs during observation of videos with hand or mouth movements, static images of hand or mouth, and white screen images in patients with high and low traits of autism, no difference between groups was observed for the white screen condition. However, participants with low traits of autism showed higher MEP amplitudes during observation of actions videos compared to static images. This result was not observed in patients with high traits of autism, which exhibited similar MEP amplitude in the observation of actions and observation of static images (Puzzo et al., 2009).

Another possibility would be the use of TMS application as a social cognitive rehabilitation tool. In a preliminary study, Sokhadze et al. (2009) rTMS applied low frequency (0.5 Hz) in the left DLPFC in 8 patients with autism, 2 times per week for 3 weeks. The results showed changes in relation to electrophysiological measures such as the reduction in the amplitude and latency of the P3 component to non-target stimuli. In addition to the normalization of P3 component, it was also observed decreased gamma frequency in frontal and parietal regions to non-target stimuli. Regarding clinical outcomes, decreased ritualistic behavior was observed as measured by scales and caregivers reported a decrease in repetitive behavior and obsessive-compulsive behaviors. Subsequent studies have replicated these results, showing the reduction of repetitive behaviors and irritability (Baruth et al., 2010; Sokhadze et al., 2010). Still on the observation of improvement in these behaviors, Casanova et al. (2012) used a protocol of rTMS (1 Hz) on CLPFC for 12, 6 weeks over left hemisphere and 6 weeks over right hemisphere. The results showed better performance on the selective attention task and improve behaviors of irritability and repetition. By using the same protocol, Sokhadze et al. (2012) also found improved performance in the error-monitoring task. In consonance with that, Sokhadze et al. (2014) have observed modulation of many electrophysiological markers and a decrease in social withdrawal scores after 18 sessions of rTMS combined with neurofeedback in ASD patients. Finally, Enticott et al. (2014) showed improvement of social skills and anxiety symptoms in 28 patients with autism (high functioning and Asperger's), after application of deep bilateral rTMS in PFC medial dorsal of 5 Hz for 15 min for 15 consecutive days. This improvement was maintained after 1 month of stimulation.

These few studies showing substantial TMS effects in social cognition in ASD might be directly related to the well-known inhibitory/excitatory neural dynamics that seems to be affected in ASD and this phenomenon seems to take place at least in part through dysfunctional glutamate and GABA neurotransmitter systems (Won et al., 2013). In fact, reduced GABA synthesis and dysfunctional GABA receptors were found in autistic brains (Fatemi et al., 2010). Abnormally low levels of glutamine and glutamate were also found in autistic children (Rolf et al., 1993). Given the fact that tDCS is known to influence glutamate and GABA concentrations depending on stimulation polarity (e.g., Filmer et al., 2014), it is reasonable to expect that this tool might be helpful in ASD rehabilitation.

Given the aforementioned advantages of tDCS relative to TMS, patients would face fewer restrictions when undergoing a treatment with the former technique, when compared to the latter. Therefore, more investigations with tDCS in ASD must be done, in order to evaluate if similar effects to the ones induced by rTMS could be achieved. Here we highlight one example of substantial tDCS modulation of structures that were also successfully modulated by TMS in previous research, and that are relevant to the treatment of ASD. Theory of mind was found to be modulated by TMS of the right temporal parietal junction (Young et al., 2010). Later, Santiesteban et al. (2012) demonstrated improvements in theory of mind and increased social ability after tDCS of the temporal parietal junction. Both experiments were performed with participants with typical development.

Childhood is arguably the most critical windows for interventions and therapy in ASD (e.g., Warren et al., 2011). The fact that the use of tDCS in children is still controversial and poorly understood (with almost no studies on safety and tolerability, e.g., Gillick et al., 2015) may be considered a drawback for its application in ASD. Nonetheless, tDCS can be applied to young adults and this is still a reasonable moment for interventions, especially if one considers that ASD symptoms will frequently still be present throughout adulthood (e.g., Tobin et al., 2014).

Lastly, a recent case study with an adult diagnosed with ASD since the age of 2 years supports all that has been proposed here. D'Urso et al. (2015) report what seems to be the first investigation to use tDCS in an ASD patient. These authors have delivered 10 daily sessions of cathodal tDCS to the left DLPFC in a patient that was non-respondent to pharmacological treatments and showed limited outcomes for behavioral interventions. The patient showed a 45% decrease in social withdrawal scores and a 58% decrease in hyperactivity (as measured in the ABC scale). The results by D'Urso et al. (2015) are very encouraging and support the need for more tDCS based investigations in all aspects of ASD, not only social cognition.

## Schizophrenia

In conditions such as schizophrenia, deficits in social cognition are known to take place and to be determinants of patients' limits in functional abilities. These deficits include impairments in Theory of Mind strategies, emotional content processing, among others (Pinkham et al., 2003). In fact, in a recent meta-analysis that investigates the relations between neurocognition (defined in this context as attention, memory, executive functions, etc.,) and social cognition in schizophrenia, Fett et al. (2011) have found that social cognition deficits were stronger determinants of patients' functional outcomes than neurocognition deficits. Another recent work has shown that these social cognition deficits are also present during remission and are only weakly correlated to neurocognition deficits (Mehta et al., 2013). These findings draw attention to the crucial role played by social cognition deficits in schizophrenia and the need to focus on social cognition in the research and treatment of these patients.

As some authors have proposed (reviewed in a meta-analysis by McGurk et al., 2007), cognitive interventions in schizophrenia are generally only able to improve functionality to a small or medium extent. As there is evidence that these social cognition impairments are present even during remission, more effort has to be put into improving this crucial aspect in this population. Therefore, non-invasive brain stimulation interventions may be very valuable and as we will present ahead, there is compelling evidence (although with only few investigations yet) that this is indeed a promising strategy.

Three meta-analyses—one on the effects of TMS in diminishing auditory hallucinations and two on the TMS use for treating negative symptoms—support that TMS is a valuable intervention for treating schizophrenia symptoms (Aleman et al., 2007; Freitas et al., 2009; Shi et al., 2014). Aleman et al. (2007) have found substantial evidence for the applicability of TMS for the treatment of auditory hallucinations in schizophrenia but no significant effect on a general index of psychotic symptoms after reviewing 10 studies. Nonetheless, Freitas et al. (2009) and Shi et al. (2014) have found significant effects of TMS on decreasing negative symptoms, a finding that supports the adequacy of this technique in treating social symptoms in schizophrenia. The rationale in most of the studies is that there is a deficit of intracortical inhibition in schizophrenia and that inhibitory non-invasive brain stimulation might improve this aspect of the disease (see Hasan et al., 2013 for a review of intracortical inhibition in schizophrenia). In fact, there is substantial evidence for a reduced density of GABA interneurons in schizophrenia and some evidence that this intracortical inhibition deficit in schizophrenia (as assessed by TMS) is significantly correlated to deficits in social cognition (Mehta et al., 2014).

tDCS has also been used in few investigation with schizophrenic patients and is showing promising outcomes. A recent systematic review by Brunoni et al. (2014) has found a number of investigations where tDCS was shown to improve auditory or visual hallucinations in schizophrenia. Two of these works have also reported a global improvement in symptoms experienced by the patients (Brunelin et al., 2012 and a case report by Palm et al., 2013). The study of Brunelin et al. (2012), for example, observed a significant reduction of auditory hallucinations (average reduction of 31%), negative and positive symptoms that was visible for up to 3 months after the tDCS sessions. Palm et al. (2013) has also found a decrease in negative symptoms and auditory hallucinations after 2 weeks of tDCS. If an improvement in negative symptoms is observed, some improvement in sociability is also expected.

The improvements reported in these studies are in accordance with what was observed in the computational models of current flow for the tDCS montages most frequently employed in this field. Brunoni et al. (2014) identified that the electrode montage used most frequently in these investigations targets the left dorsolateral prefrontal cortex (an area associated with negative symptoms in schizophrenia) and that the current generally flows through deeper structures involved in schizophrenic symptoms (as the cingulated cortex, insula, basal ganglia, and hippocampus).

All the evidence mentioned here supports the need and relevance of more studies on brain stimulation and social cognition in schizophrenia. As we have argued above, social cognition is impaired in schizophrenia and it seems to be a stronger determinant of patients' functional outcomes than neurocognition (Fett et al., 2011). Therefore, it is surprising and noteworthy how there are still very few investigations of the non-invasive brain stimulation effects in social functioning in schizophrenic These techniques seem to have an important role as tools to modulate positive and negative symptoms in schizophrenia. This is particularly relevant when considering that many participants are drug resistant or present medication refractory schizophrenia. In these cases, non-pharmacological interventions as TMS and tDCS might be great alternatives.

## Williams syndrome

Williams syndrome (WS) is a rare neurodevelopmental disorder caused by a multiple gene deletion on chromosome 7q11.23 (Korenberg et al., 2000). Apart from a set of medical, physical, and cognitive features (including mild to moderate intellectual disability), individuals with the disorder are commonly described as having the opposite social profile of individuals with ASD (Karmiloff-Smith et al., 2012). Indeed, several studies have reported exacerbated interest in social stimuli and excessive social approach (including to strangers) as remarkable features present from early infancy into adulthood—a tendency termed *hypersociability* (Jones et al., 2000; Mervis et al., 2001; Laing et al., 2002; Riby and Hancock, 2008, 2009). However, these seemingly endearing personality features often pose important threats to these individuals, putting them at risk in unsupervised social interactions. For instance, studies showed that persons with WS tend to rate unfamiliar faces as significantly more approachable than age-matched controls (Jones et al., 2000; Martens et al., 2009). Furthermore, a recent study found that these individuals are more likely to consider approaching and ultimately choose to approach untrustworthy faces significantly more than controls matched in chronological age (Martens et al., 2012). These same behavioral tendencies studied in laboratory settings are reported to occur in everyday interactions, with a significant negative impact for individuals with this syndrome. In particular, they experience greater rates of sexual abuse when compared to samples with intellectual disability (Rosner et al., 2004). In a recent paper by Fisher et al. (2013) parents of individuals with WS reported higher levels of social vulnerability—particularly lower risk awareness by their children—than parents of individuals with ASD or Down syndrome. Consistent with these reports, some authors highlight how hypersociability goes alongside difficulties in regulating social behaviors (Jawaid et al., 2012; Riby et al., 2014), drawing attention to the deleterious effects of hypersociability for individuals with WS and their families.

Several hypotheses have been advanced to explain this trait, namely the amygdala hypothesis and the prefrontal hypothesis (Jawaid et al., 2008; Capitão et al., 2011). The former suggests that structural or functional alterations in the amygdala might account for the difficulties in recognizing threat—particularly in social interactions—, while the latter proposes that despite being able to detect social threat, individuals with WS are unable to inhibit approach behaviors due to prefrontal impairments. The results reported so far seem to suggest that these hypotheses might be complementary, rather than mutually exclusive (Mimura et al., 2010; Capitão et al., 2011). In this line, Meyer-Lindenberg et al. (2005) demonstrated the joint activation of the amygdala along with dorsolateral prefrontal cortex (DLPFC), medial prefrontal cortex (MPFC), and orbitofrontal cortex (OFC) in a threatening face matching task in healthy controls. In contrast, this pattern of joint activation was absent in a select sample of WS with intact cognitive abilities. More specifically, OFC was not selectively recruited and path analysis further showed that this region was not functionally connected to the amygdala—a result that is consonant with the strong social disinhibition seen in WS. In addition, abnormal interactions were found between MPFC and DLPFC in WS, which were consistent with a possible compensatory mechanism for the disrupted OFC-amygdala circuit (Meyer-Lindenberg et al., 2005). In a subsequent study with the same sample, Munoz et al. (2010) reported further evidence of abnormal circuitry involved in the processing of social vs. non-social threat in WS, reinforcing these regions as likely involved in the emergence of hypersociability.

Together, these findings open new avenues for the use of neuromodulation, as the aforementioned brain regions afford possible target areas aimed at improving social cognition and reducing hypersociable tendencies in WS. So far, only sparse behavioral interventions (mostly case studies) have been implemented with these goals in mind, with inconsistent success (Klein-Tasman and Albano, 2007; Phillips and Klein-Tasman, 2009; Jawaid et al., 2012). Importantly, no studies have employed either TMS or tDCS in this clinical population. We argue that the successful use of these non-invasive and generally safe techniques in several other neurodevelopmental disorders (particularly those cited in this paper) provides encouragement for future studies to test its therapeutic potential in WS.

## Noteworthy issues in brain stimulation

The current review focuses on two of the most popular brain stimulation techniques: tDCS and TMS. These techniques differ in dynamics and mechanisms of action to a great extent. tDCS is associated with moderate modulation of the voltage gradient between the intracellular and extracellular medium, while TMS can induce action potentials. These techniques also differ in the focality of the stimulation and its potential outcomes. For recent reviews of parameters and mechanisms of action we refer to Stagg and Nitsche (2011) and Filmer et al. (2014) for tDCS and Pascual-Leone et al. (2000) and Miniussi and Rossini (2011) for TMS.

For the readers that are not familiar with non-invasive brain stimulation, we must highlight the safety of these techniques when standard parameters are used. For tDCS, extensive data suggest that this is a safe procedure that involves mild and transient adverse effects (Nitsche et al., 2008; Brunoni et al., 2012). In a systematic review of tDCS studies, Brunoni et al. (2012) found that 56% of the investigations mentioned adverse effects but these were limited to itching or tingling under the electrodes, headache, and discomfort. These adverse effects were also present in participants receiving sham tDCS, thus suggesting that the major cause of side effects may not be the current itself. Recent studies showed that tDCS does not induce elevations of a neuronal damage marker (neuron-specific enolase) and no edemas in humans and no maladaptive functional or structural changes were observed in evaluations using EEG and fMRI (Nitsche et al., 2004, 2008; Iyer et al., 2005). It is still not clear if repeated tDCS sessions during weeks and months can lead to undesirable side effects and more research on that matter is advised.

Although in some cases TMS might be associated with more severe adverse effects than tDCS, generally its adverse effects are mild and transient and the technique is considered safe when standard parameters are employed (e.g., Rossi et al., 2009). In general terms TMS protocols may be divided between the ones that use isolated single pulses of stimulation, the ones that use paired pulses separated by very brief intervals and the ones that use repetitive pulses at one specific temporal frequency that might range from a few seconds to minutes. Amongst these, repetitive TMS protocols are the ones with more long lasting effects and more significant therapeutic potential. It is also the stimulation modality that is associated with more significant adverse effects, and this is why many guidelines suggest it should be delivered in hospital facilities (Lefaucheur et al., 2014).

The ease of use, portability and minimal adverse effects associated with tDCS might be considered one of its advantages over TMS. In fact, in the field of pain treatment research, a systematic review by Zaghi et al. (2009) concluded that when compared to other neuromodulatory techniques (such as repetitive TMS or EDCS), tDCS is the most cost-effective treatment for central pain (considering treatments shorter than 5 years). Nonetheless, each technique has its strengths and possible drawbacks. This must be considered carefully when planning each investigation. The functional and spatial focality of the two techniques is frequently a matter of debate, and in many cases there is no easy answer for which technique to employ. In fact, more research comparing these techniques is also advised.

Lastly, as the clinical conditions reviewed here are more frequently diagnosed first in children and adolescents, a brief mention to possible peculiar effects of non-invasive brain stimulation in the developing brain must be made. Until recently there were only few works on the effects of tDCS and TMS in children and adolescents. This was probably determined at least in part by ethical concerns, given the fact that the developing brain could be affected in non-intuitive ways by these interventions. Recent advances in our understanding of mechanisms of action and outcomes of non-invasive brain stimulation techniques, paired with a few successful investigations in children are changing this scenario.

A recent review of TMS studies in children has suggested that this intervention is well tolerated and promising (Frye et al., 2008). Nonetheless the authors have suggested that specific guidelines need to be created for children. The same is true for tDCS. Some recent works have suggested that tDCS might be effective and well tolerated in children (Minhas et al., 2012; Andrade et al., 2014), but more investigations of this subject are still lacking. It is also important to note that in many cases, a rationale that was proven to be successful for stimulation in adults might not turn out to have the same outcomes in the developing brain and more research in this population is still needed.

## Conclusions and future directions

This article presents evidence that neuromodulation techniques may become important interventional tools in social deficits. Research focused on the understanding of the mechanisms of action of these techniques as well as on ways to direct their effects to enhance social cognition deficits will open new avenues in what we are calling Social Plasticity. As shown, there are few studies directly evaluating the effects of tDCS and TMS in populations such as autism, schizophrenia and WS. As regards to the social cognition deficits, these techniques are still in their infancy. With that, a research agenda in this area is indispensable. As central aspects, it is necessary to standardize procedures like the use of common assessment tools. It is also necessary to initially investigate the short-term effects of these techniques (for example, single session stimulation) and then move forward with investigations on the lasting effects of these tools. This care is critical considering the potential that these techniques have in the promotion of brain plasticity. Another key aspect relates to the large heterogeneity of the manifested symptoms. Taking autism as an example, the great diversity of manifestations within the spectrum makes you take precautions in the translation of knowledge from one study to another. Based on the studies of cognitive neuroscience, it is clear that non-invasive neuromodulation techniques are already part of the toolkit box of both research and rehabilitation domains. With the first evidences presented in this review, it is flagrant the need to insert these techniques in the social neuroscientist arsenal particularly focused on rehabilitation and promotion of social plasticity.

## Author contributions

All authors contributed to manuscript writing and approved the final version of the manuscript for submission.

### Conflict of interest statement

The authors declare that the research was conducted in the absence of any commercial or financial relationships that could be construed as a potential conflict of interest.
